# Comparing the impact of transcatheter ASD closure on echocardiographic indices in adults below and above 50 years

**DOI:** 10.1186/s44156-025-00074-3

**Published:** 2025-05-05

**Authors:** Reza Kiani, Parisa Firoozbakhsh, Negar Dokhani, Azin Alizadehasl, Hooman Bakhshandeh, Ata Firouzi, Ali Zahedmehr, Mahnaz Daneshzadeh

**Affiliations:** 1 Cardiovascular Intervention Research Center, Rajaie Cardiovascular Medical and Research Center, Iran University of Medical Sciences, Tehran, Iran; 2 Cardiac Primary Prevention Research Center, Cardiovascular Diseases Research Institute, Tehran University of Medical Sciences, Tehran, Iran; 3 Cardio-Oncology Research Center, Rajaie Cardiovascular Medical and Research Center, Iran University of Medical Sciences, Tehran, Iran; 4 Rajaie Cardiovascular Medical and Research Center, Iran University of Medical Sciences, Tehran, Iran; 5 Rajaie Cardiovascular Medical and Research Center, School of Medicine, Iran University of Medical Sciences, Tehran, Iran

**Keywords:** Heart Defects, Congenital, Heart Septal Defects, Atrial, Aged, Transcatheter device closure, Echocardiography

## Abstract

**Background:**

Transcatheter device closure is the method of choice for the closure of secundum atrial septal defects (ASD) with appropriate anatomic characteristics, leading to symptomatic relief, increased survival rates, cardiac remodeling, and improved cardiac function.

**Objective:**

Assessing the impact of transcatheter ASD closure on echocardiographic indices and comparing them between individuals younger and older than 50.

**Method:**

In this retrospective cohort study, 240 patients with isolated secundum ASD and complete documentation and follow-up data who underwent transcatheter device closure between 2015 and 2019 were included. Demographic, peri-procedural, and echocardiographic findings were compared before and after the procedure and among two age groups.

**Results:**

A total of 240 patients (68% female, 44% younger than 50) with a median age of 51 underwent transcatheter ASD closure. ASD closure led to a significant decline in the size of four cardiac chambers and systolic pulmonary arterial pressure (SPAP), in addition to a significant improvement in biventricular systolic function, LV diastolic function, and valvular insufficiencies. Although patients aged 50 and older had worse LV diastolic and RV systolic function, in addition to larger RV size and bi-atrial dimensions at the baseline, the extent of improvement of these parameters among them was significantly more pronounced than those younger than 50. There were no significant differences in the extent of the decline in SPAP between the two groups.

**Conclusion:**

Transcatheter ASD device closure is a beneficial procedure with high success rates and low complication rates among older individuals, eventually leading to improvements in cardiac form and function.

**Supplementary Information:**

The online version contains supplementary material available at 10.1186/s44156-025-00074-3.

## Background

Atrial septal defect (ASD), with an estimated prevalence of 0.88 per 1000 adults, is the most common congenital heart disease (CHD) diagnosed in adulthood, accounting for 25–30% of adult-onset heart diseases [1, 2]. The majority of patients may remain asymptomatic until their second to fourth decades of life and present in adulthood with fatigue, exertional dyspnea, and palpitations that worsen as the age advances [3]. Delayed diagnosis and untreated lesions may lead to the development of ASD complications, including atrial arrhythmias (atrial flutter and atrial fibrillation), paradoxical emboli, and cerebral abscesses. A continuous left-to-right shunt may also lead to right ventricular (RV) volume overload and subsequent RV failure, accompanied by pulmonary vascular remodeling and pulmonary hypertension (PH), which can reverse the shunt direction and lead to Eisenmenger syndrome [4].

ASDs with evidence of right ventricular volume overload and no pulmonary arterial hypertension or left ventricular (LV) disease are indicated for closure [5]. ASD closure can be performed surgically or through a transcatheter approach. Transcatheter device closure, first introduced by King and colleagues in 1974, is considered the method of choice for the closure of secundum ASDs with appropriate anatomic characteristics [6, 7]. It is a safe and efficient method with similar efficacy and mortality rates to surgical closure, in addition to lower complication rates, shorter hospital stays, and a faster recovery period [8, 9]. Defect closure leads to symptomatic relief, improved functional class, exercise capacity, quality of life, and increased survival rates in all age groups [10, 11, 12]. It is also associated with right and left cardiac remodeling that appears as reductions in left atrial and right heart volumes, improvements in left and right ventricular function, and increases in left ventricular filling [6, 13].

Since the extent of cardiac remodeling decreases and patient morbidity increases as the age at the time of closure advances, and the 5-year re-intervention rate is also higher in the transcatheter approach compared to the surgery, there is an ongoing debate regarding the indications of ASD closure in older patients, especially in asymptomatic or mildly symptomatic individuals and those with normal pulmonary pressure [14], and the main question is whether they would benefit from defect closure or not [6, 9]. Recent studies have not yet found a definitive answer to this question. In the current study, we aimed to evaluate the effect of transcatheter ASD closure on cardiac remodeling and improvement of echocardiographic indices in ASD patients older than 50 years old and to compare our findings with patients younger than 50.

## Material and method

### Study design and population

In this retrospective cohort study, all adult patients with isolated secundum ASD who were indicated for defect closure and underwent transcatheter device closure between April 2015 and March 2019 at Rajaie Cardiovascular Medical and Research Center, the largest tertiary care center for cardiovascular disorders in Tehran, Iran, were considered eligible. Patients were divided into two groups based on age: Group 1 consisted of patients younger than 50, and Group 2 consisted of those aged 50 and above.

Indications of defect closure included the presence of a left-to-right shunt, RA, and/or RV enlargement with pulmonary to systemic blood flow ratio (Q_p_/Q_s_) ≥ 1.5:1 or platypnea-orthodeoxia resulting from ASD. Before the procedure, each patient underwent transthoracic echocardiography (TTE) and transesophageal echocardiography (TEE). The patients’ follow-up after the procedure was conducted using TTE within a 6 ± 1 month period. All baseline, intraoperative, and follow-up echocardiographic evaluations were performed by two echocardiologists who were specifically trained in ASD assessment. They both followed standardized imaging protocols and interpretation criteria, ensuring uniformity and reliability in data acquisition and analysis. The patient’s echocardiographic findings before and after the procedure were compared. Furthermore, patients’ demographic, peri-procedural, and echocardiographic findings were compared among two age groups.

### Device closure

All ASD patients underwent a TEE before the procedure to assess the size of the defect and also the direction of the shunt. Additionally, a TTE was performed to provide a general cardiac evaluation, evaluate the presence of other structural heart abnormalities, and determine the possibility of catheter device closure. If the patients were deemed eligible for transcatheter device closure, the procedure was initiated by an expert interventional cardiologist, an echocardiologist responsible for performing the TEE during the procedure, and the catheterization lab team. The procedure commenced with a femoral vein access. Once the catheter was inserted into the right atrium (RA) and the left-to-right shunt was re-evaluated, the appropriate choice for device type was made between Amplatzer™ Septal Occluder (Abbott, Chicago, IL, USA) and the Figulla^®^ ASD Occluder (Occlutech, Jena, Germany), considering the defect size, rim adequacy, septal morphology, and device availability at the time of the procedure. Throughout the procedure, the echocardiologist continuously assessed the accuracy of device placement, checked for any residual shunts, and monitored for potential adverse effects.

### Follow-up

After device closure, the patients were placed on a six-month course of aspirin treatment, followed by a TTE to assess residual shunting and any potential adverse effects. If any patient needed any procedure that could potentially lead to bacteremia within six months after defect closure, antibiotics were prescribed for prophylaxis against infective endocarditis.

### Statistical analysis

Numerical data were presented as medians (Q_1_-Q_3_), while categorical data were represented by frequency (percent). To assess the normal distribution of data, the One-sample Kolmogorov-Smirnov test was used. Furthermore, to compare the variables before and after ASD device closure, a Wilcoxon signed-ranks test was applied. To compare variables across two age groups, Pearson’s chi-square test (or Fisher’s exact test) was used for quantitative data, and the Mann-Whitney U test was applied for qualitative data. The significance level was defined at *P* < 0.05. The data analysis was carried out using IBM SPSS Statistics for Windows, version 22 (IBM Corp., Armonk, N.Y., USA). Charts were prepared using Microsoft Excel 2016.

### Ethical considerations

This study complies with the Declaration of Helsinki and was approved by the ethics committee of Rajaie Cardiovascular Medical and Research Center (Ethical Approval Code: IR.IUMS.FMD.REC.1400.266). The current study was conducted in accordance with the latest relevant guidelines and regulations, and all data gathered during this study were confidential. After providing a detailed explanation of the procedure and the aims of the study, written and verbal informed consent was obtained from all participants prior to the catheter device closure and also for using their data in the current study.

## Results

### Patient characteristics

Among the 1125 individuals who underwent device closure for secundum ASD between April 2015 and March 2019, only 240 patients had isolated secundum ASD, along with complete documentation and follow-up data, and were included in the current study, of whom 163 (68%) were female. The median age (Q_1_-Q_3_) of the patients was 51 (36–59) years, with 56% (135 patients) being 50 years or older. Common cardiovascular risk factors, including hypertension, diabetes mellitus, and smoking were significantly more prevalent among patients older than 50. Details of the demographic and clinical characteristics of the study population are demonstrated in Table 1.

**Table 1:** Baseline demographic and clinical characteristics of the study population

	Total (*n* = 240)	Age < 50 years (*n* = 105)	Age ≥ 50 years (*n* = 135)	*P*-value
**Demographic characteristics**				
Age (years), median [Q_1_-Q_3_]	51 [36–59]	34 [27.5–42]	58 [53–63]	**< 0.001**
Sex (Female), *n* (%)	163 (68%)	72 (68.5%)	91 (67.4%)	0.848
**Cardiovascular risk factors**				
Hypertension, *n* (%)	79 (33%)	25 (24%)	54 (40%)	**0.008**
Dyslipidemia, *n* (%)	27 (11%)	18 (17%)	9 (7%)	**0.011**
Diabetes mellitus, *n* (%)	22 (9%)	2 (2%)	20 (15%)	**0.001**
Smoking, *n* (%)	11 (5%)	0 (0%)	11 (8%)	**0.003**
History of CVA/ TIA, *n* (%)	7 (3%)	1 (1%)	6 (4%)	0.14

**P-values less than 0.05 are shown in bold Abbreviations**: CVA:Cerebrovascular accident, SD: Standard deviation, TIA: Transient ischemic attack

### Device and defect characteristics

The participants had an ASD with a median size of 1.8 × 1.4 cm, median (Q_1_-Q_3_) Q_p_/Q_s_ of 1.9 (1.7–2.2), and a median (Q_1_-Q_3_) implanted device size of 21 mm (18–27 mm) with no significant differences in two age groups. The two types of ASD occluder devices used were Amplatzer™ Septal Occluder (Abbott, Chicago, IL, USA) and the Figulla^®^ ASD Occluder (Occlutech, Jena, Germany), which were implanted in 3% and 97% of the participants, respectively. Details of the anatomic characteristics of the defect and the occluder devices are provided in Table 2.

**Table 2:** Characteristics of the defect anatomy and the implanted occluder device

	Total population	Age < 50 years (*n* = 105)	Age ≥ 50 years (*n* = 135)	*P*-value
**Qp/Qs**, **median [Q**_**1**_**-Q**_**3**_**]**	1.9 [1.7–2.2]	1.9 [1.7-2]	1.8 [1.7–2.4]	0.85
**Defect size (cm)**, **median [Q**_**1**_**-Q**_**3**_**]**				
Largest diameter	1.8 [1.5–2.1]	1.7 [1.5-2]	1.8 [1.6–2.2]	0.21
Smallest diameter	1.4 [1.2–1.6]	1.4 [1.2–1.6]	1.4 [1.2–1.6]	0.23
**Rim (cm)**, **median [Q**_**1**_**-Q**_**3**_**]**				
Anteroinferior rim	1 [0.7–1.3]	1 [0.7–1.3]	1 [0.8–1.4]	0.15
Posteroinferior rim	1.1 [0.8–1.4]	1.1 [0.8–1.4]	1.2 [0.8–1.6]	0.11
Anterosuperior rim	0.7 [0.4–1.2]	0.7 [0.4-1]	0.7 [0.4–1.3]	0.55
Posterosuperior rim	1 [0.8–1.3]	1 [0.7–1.2]	1 [0.8–1.4]	**0.02**
IVC rim	1.3 [1-1.6]	1.3 [1-1.6]	1.3 [0.9–1.6]	0.44
SVC rim	1.1 [0.8–1.4]	1.2 [0.8–1.4]	1.1 [0.7–1.4]	0.59
**Device size (mm)**, **median [Q**_**1**_**-Q**_**3**_**]**	21 [18–27]	21 [18–27]	24 [18–27]	0.71
**Device type**, ***n*(%)**				**0.019**
Amplatzer Septal Occluder	7 (3%)	0 (0%)	7 (5%)	
Figulla ASD Occluder	233 (97%)	105 (100%)	128 (95%)	

**P-values less than 0.05 are shown in bold Abbreviations**: ASD: Atrial septal defect, IVC: Inferior vena cava, Qp/Qs: Pulmonary to systemic blood flow ratio, SVC: Superior vena cava

### Echocardiographic assessment of left ventricular (LV) parameters

Patients had larger LV sizes prior to ASD closure, and in the follow-up echocardiography performed six months after the defect closure, there was a significant reduction in LV size compared to the baseline echocardiography. Additionally, other echocardiographic LV parameters, including LV ejection fraction (LVEF) and LV diastolic function, showed significant improvements following ASD device closure (Table 3).

**Table 3:** Baseline and follow-up echocardiographic findings of the total study population

	Baseline	Follow-up	*P*-value
**LV parameters**			
LV size (mm), mean [min-max]	45 [40–50]	40 [37–45]	**< 0.001**
LV size category, *n* (%)			**< 0.001**
Normal	220 (92%)	234 (97%)	
Mild enlargement	14 (6%)	5 (2%)	
Moderate enlargement	6 (2%)	1 (1%)	
LVEF, *n* (%)			**< 0.001**
Normal	86 (36%)	165 (69%)	
Mild dysfunction	138 (57%)	75 (31%)	
Moderate dysfunction	16 (7%)	0 (0%)	
LV diastolic dysfunction, *n* (%)			**< 0.001**
Normal	139 (58%)	199 (83%)	
Mild dysfunction	90 (37%)	39 (16%)	
Moderate dysfunction	9 (4%)	2 (1%)	
Severe dysfunction	2 (1%)	0 (0%)	
**RV parameters**			
RV size (mm), mean [min-max]	34 [30–38]	30 [28–34]	**< 0.001**
RV size category, *n* (%)			**< 0.001**
Normal	111 (46%)	172 (72%)	
Mild enlargement	65 (27%)	57 (24%)	
Moderate enlargement	53 (22%)	11 (4%)	
Severe enlargement	11 (5%)	0 (0%)	
RV function, *n* (%)			**< 0.001**
Normal	91 (38%)	187 (78%)	
Mild dysfunction	131 (55%)	53 (22%)	
Moderate dysfunction	18 (7%)	0 (0%)	
SPAP (mmHg), median [Q_1_-Q_3_]	34 [30–38]	27 [24–32]	**< 0.001**
SPAP category, *n* (%)			**< 0.001**
Normal	132 (55%)	196 (82%)	
Top normal	56 (23%)	27 (11%)	
Mild PH	45 (19%)	16 (6%)	
Moderate PH	7 (3%)	1 (1%)	
**Atrial parameters**			
LAVi (mL/m2), mean [min-max]	34 [29–39]	30 [25–34]	**< 0.001**
LA size category, *n* (%)			**< 0.001**
Normal	131 (55%)	187 (78%)	
Mild enlargement	74 (31%)	47 (19%)	
Moderate enlargement	27 (11%)	5 (2%)	
Severe enlargement	8 (3%)	1 (1%)	
RAVi (mL/m2), mean [min-max]	30 [27–35]	26 [24–30]	**< 0.001**
RA size category, *n* (%)			**< 0.001**
Normal	98 (41%)	174 (72%)	
Mild enlargement	88 (37%)	52 (22%)	
Moderate enlargement	42 (17%)	12 (5%)	
Severe enlargement	12 (5%)	2 (1%)	
**Valvular parameters**			
MR, *n* (%)			**< 0.001**
No MR	13 (5%)	196 (82%)	
Mild MR	207 (86%)	44 (18%)	
Moderate MR	20 (9%)	0 (0%)	
TR, *n* (%)			**< 0.001**
No TR	15 (6%)	121 (50%)	
Mild TR	177 (74%)	118 (49%)	
Moderate TR	48 (20%)	1 (1%)	
AR, *n* (%)			**0.033**
No AR	183 (76%)	190 (79%)	
Mild AR	55 (23%)	49 (20%)	
Moderate and severe AR	2 (1%)	1 (1%)	

**P-values less than 0.05 are shown in bold Abbreviations**: AR: Aortic regurgitation, LA: Left atrium, LAVi: Left atrial volume index, LV: Left ventricle, LVEF: Left ventricular ejection fraction, MR: Mitral regurgitation, PH: Pulmonary hypertension, RA: Right atrium, RAVi: Right atrial volume index, RV: Right ventricle, SPAP: Systolic pulmonary artery pressure, TR: Tricuspid regurgitation

**Fig. 1:**
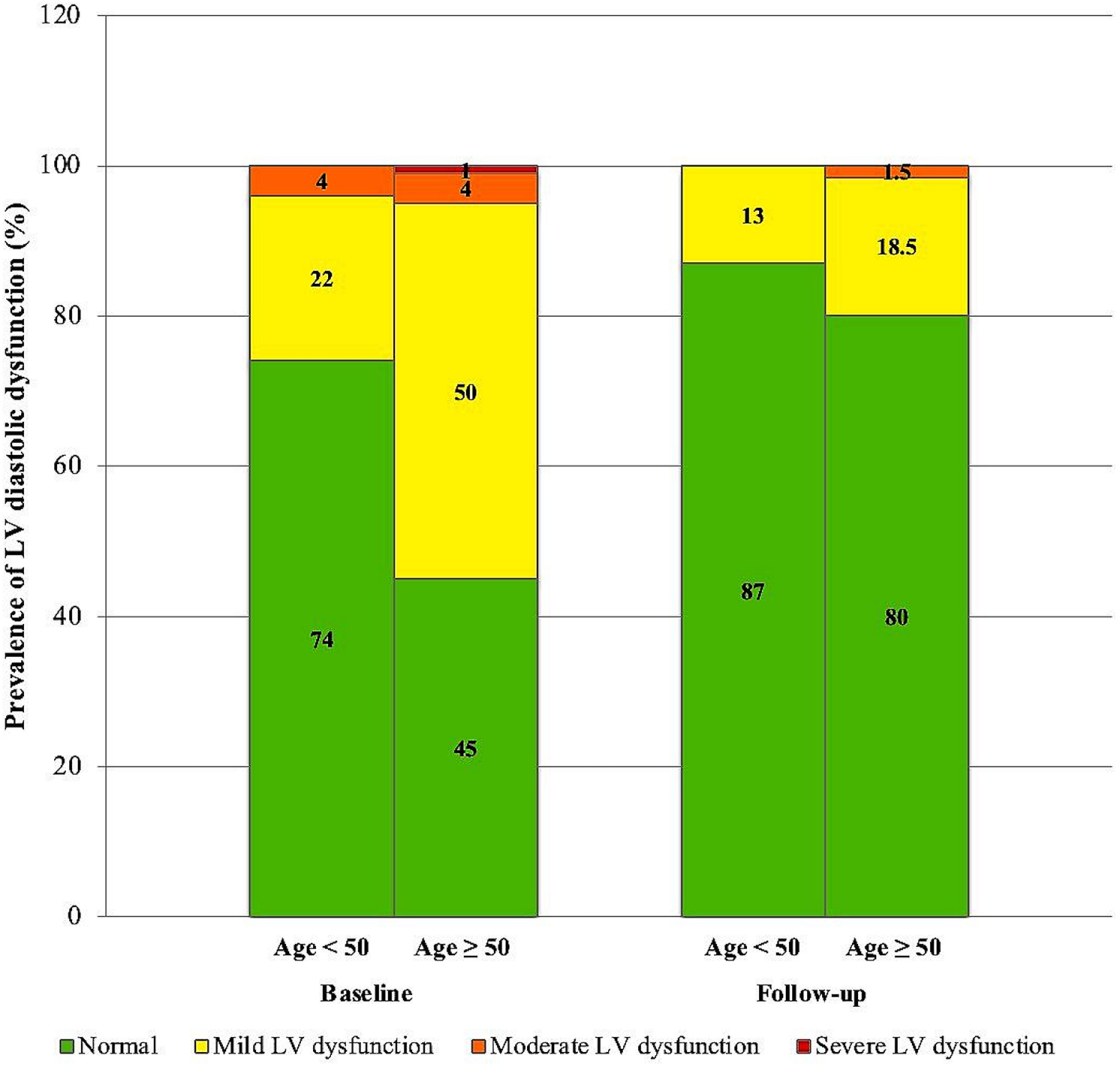
Comparing the prevalence of LV diastolic dysfunction before and after the defect closure in two age groups. LV: left ventricle

The extent of changes in LV size and function after the procedure did not significantly differ between the two age groups. Concerning left ventricular diastolic function, although LV diastolic dysfunction was significantly more prevalent among patients older than 50 at baseline (Supplementary Table S1), the degree of improvement in LV diastolic function following device closure was significantly more prominent in individuals older than 50 compared to those under 50 (Table 4; Fig. 1).

**Table 4:** Changes in echocardiographic findings in the follow-up echocardiography compared to the baseline

	Age < 50 years (*n* = 105)	Age ≥ 50 years (*n* = 135)	*P*-value
**LV parameters**			
LV size (mm), median [Q_1_-Q_3_]	3 [2–5]	3 [2–5]	0.077
LV enlargement, *n* (%)			0.565
2 stages worsen	0 (0%)	2 (2%)	
No change	96 (91%)	122 (90%)	
1 stage better	9 (9%)	10 (8%)	
2 stages better	0 (0%)	1 (1%)	
LVEF, *n* (%)			0.118
1 stage worsen	2 (2%)	3 (2%)	
No change	67 (64%)	70 (52%)	
1 stage better	35 (33%)	61 (45%)	
2 stages better	1 (1%)	1 (1%)	
LV diastolic dysfunction, *n* (%)			**< 0.001**
No change	88 (84%)	81 (60%)	
1 stage better	17 (16%)	54 (40%)	
**RV parameters**			
RV size (mm), median [Q_1_-Q_3_]	2 [0–5]	4 [2–5]	**< 0.001**
RV enlargement, *n* (%)			**0.003**
No change	61 (58%)	54 (40%)	
1 stage better	43 (41%)	76 (56%)	
2 stages better	1 (1%)	5 (4%)	
RV function, *n* (%)			**< 0.001**
No change	79 (75%)	47 (35%)	
1 stage better	26 (25%)	88 (65%)	
SPAP (mmHg), median [Q_1_-Q_3_]	6 [3–10]	7 [3–10]	0.621
SPAP category, *n* (%)			0.302
2 stages worsen	3 (3%)	3 (3%)	
1 stage worsen	1 (1%)	7 (5%)	
No change	67 (64%)	69 (51%)	
1 stage better	22 (21%)	34 (25%)	
2 stages better	12 (11%)	21 (16%)	
3 stages better	0 (0%)	1 (1%)	
**Atrial parameters**			
LAVi (mL/m2), median [Q_1_-Q_3_]	3 [2–4]	4 [2–5]	**< 0.001**
LA enlargement, *n* (%)			**< 0.001**
1 stage worsen	2 (2%)	0 (0%)	
No change	78 (74%)	75 (56%)	
1 stage better	24 (23%)	52 (38%)	
2 stages better	1 (1%)	8 (6%)	
RAVi (mL/m2), median [Q_1_-Q_3_]	3 [2–4]	4 [3–6]	**< 0.001**
RA enlargement, *n* (%)			**< 0.001**
No change	84 (80%)	49 (37%)	
1 stage better	21 (20%)	69 (51%)	
2 stages better	0 (0%)	15 (11%)	
3 stages better	0 (0%)	1 (1%)	
**Valvular parameters**			
MR, *n* (%)			0.113
No change	22 (21%)	18 (13%)	
1 stage better	82 (78%)	115 (85%)	
2 stages better	1 (1%)	2 (2%)	
TR, *n* (%)			0.807
1 stage worsen	0 (0%)	1 (1%)	
No change	39 (37%)	47 (35%)	
1 stage better	66 (63%)	86 (64%)	
2 stages better	0 (0%)	1 (1%)	
AR, *n* (%)			0.563
No change	105 (100%)	134 (99%)	
1 stage better	0 (0%)	1 (1%)	

**P-values less than 0.05 are shown in bold Abbreviations**: AR: Aortic regurgitation, LA: Left atrium, LAVi: Left atrial volume index, LV: Left ventricle, LVEF: Left ventricular ejection fraction, MR: Mitral regurgitation, RA:Right atrium, RAVi: Right atrial volume index, RV: Right ventricle, SPAP: Systolic pulmonary artery pressure, TR: Tricuspid regurgitation

### Echocardiographic assessment of right ventricular (RV) parameters

ASD closure resulted in a significant reduction in RV size and a significant enhancement in RV function among the study participants. The median systolic pulmonary arterial pressure (SPAP) and the prevalence of top normal, mild, and moderate pulmonary hypertension were significantly reduced after the procedure (Table 3).

**Fig. 2:**
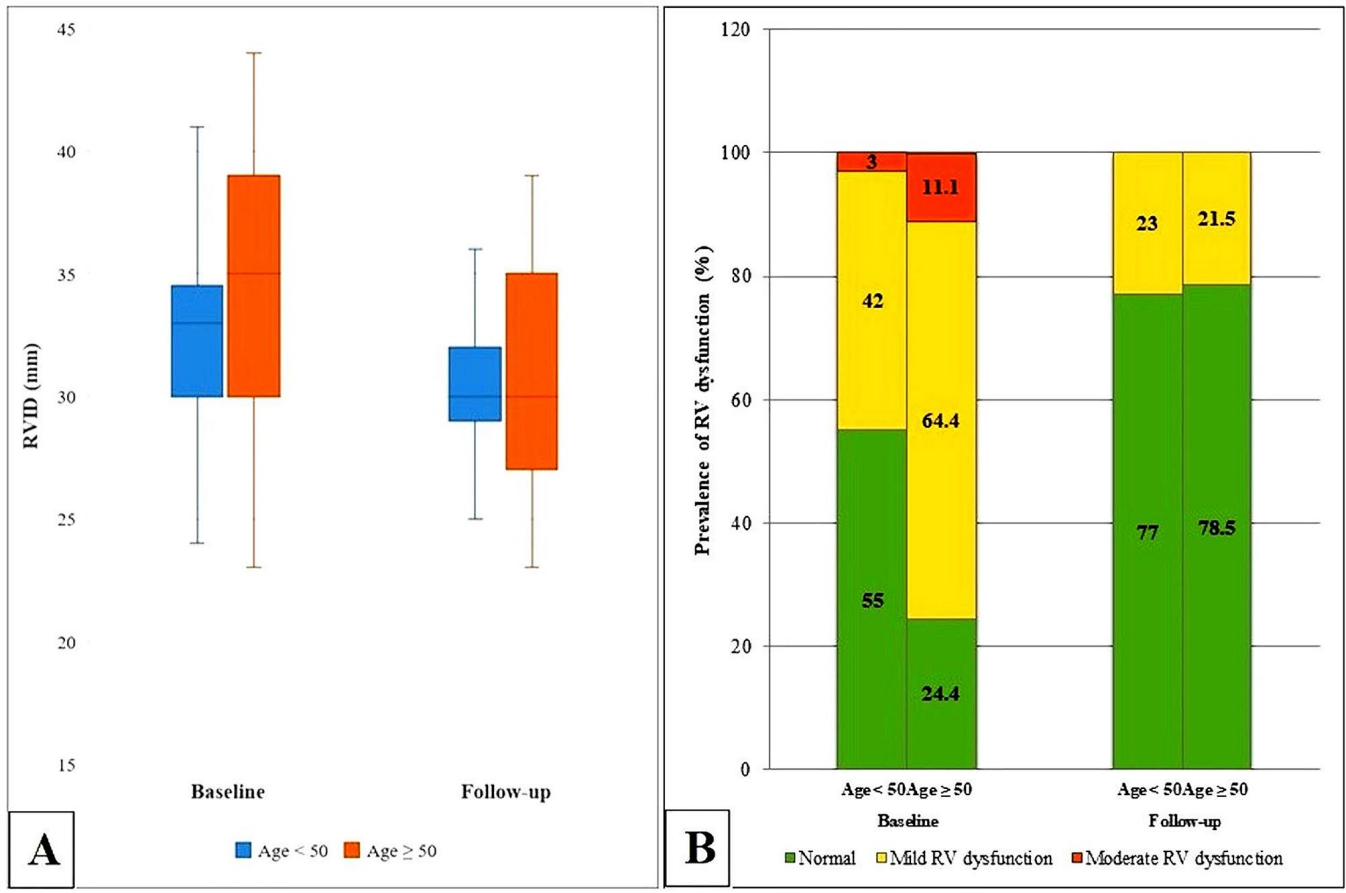
Comparing the (**A**) End-diastolic RVID and (**B**) Prevalence of RV dysfunction before and after the procedure in two age groups. RV: Right ventricle, RVID: Right ventricular internal diameter

Patients aged 50 and older had larger RV sizes and worse RV function at the baseline than those younger than 50 (Supplementary Table S1). However, their reduction in RV size and improvement in RV function following the defect closure were significantly more pronounced than the patients younger than 50 (Table 4; Fig. 2).

The degree of SPAP reduction was not significantly different in the two age groups (Table 4), and patients aged 50 and older had significantly higher SPAP values and a greater prevalence of pulmonary hypertension, both at baseline and follow-up echocardiography, compared to individuals younger than 50 (Supplementary Table S1).

### Echocardiographic assessment of atrial parameters

ASD patients had larger left and right atrium sizes and a higher prevalence of atrial enlargement at the baseline. However, after ASD closure, a significant reduction was observed in their left atrial volume index (LAVi) and right atrial volume index (RAVi), alongside a significant decrease in the prevalence of both right and left atrial enlargement (Table 3).

**Fig. 3:**
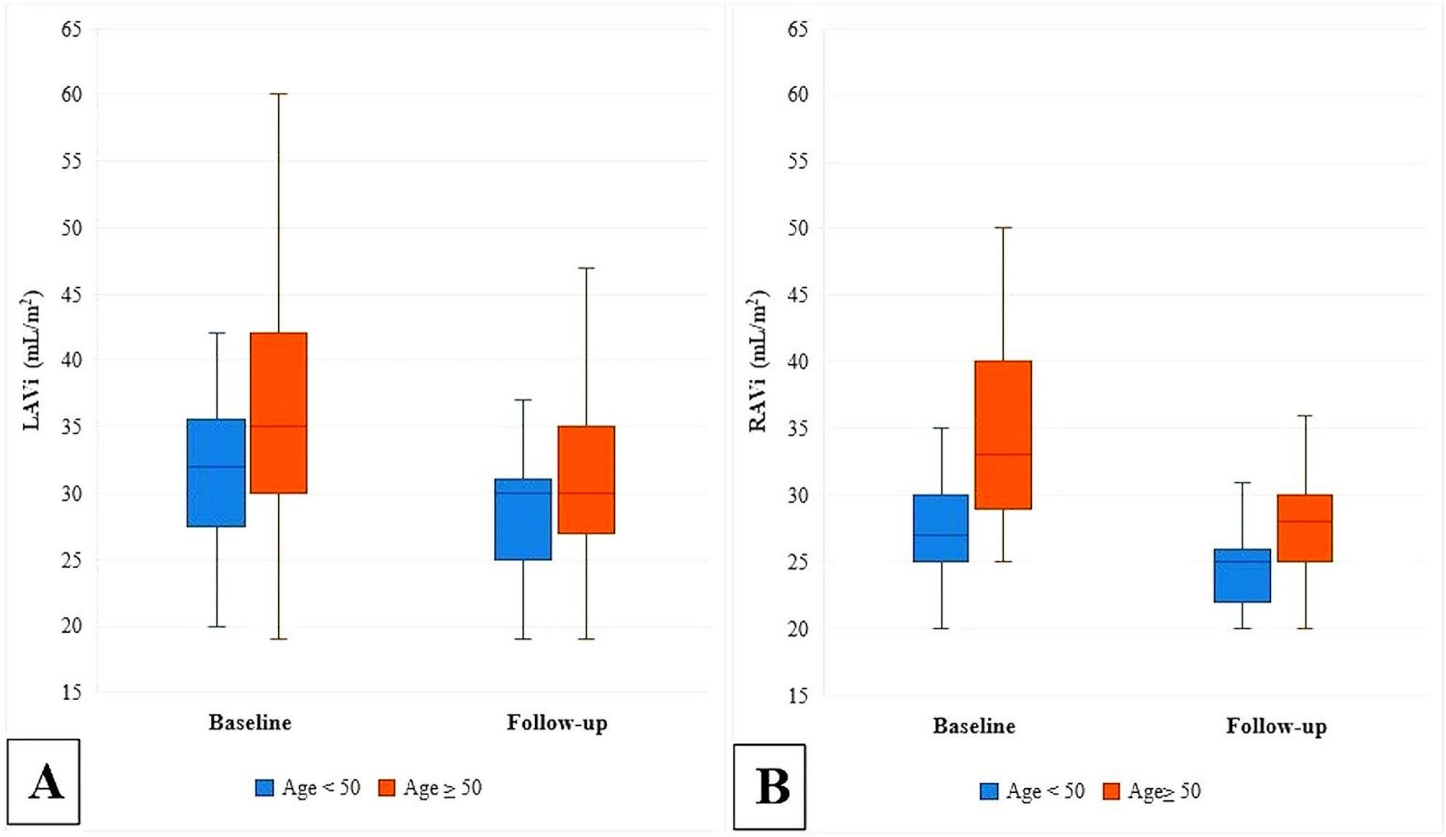
Comparing the (**A**) LAVi, and (**B**) RAVi before and after the procedure in two age groups. LAVi: Left atrial volume index, RAVi: Right atrial volume index

Although patients aged 50 and older had significantly larger atrial dimensions and a significantly higher prevalence of atrial enlargement at the baseline (Supplementary Table S1)), their extent of reduction in atrial sizes and prevalence of atrial enlargement following ASD closure was significantly more prominent than the patients younger than 50 (Table 4; Fig. 3).

### Echocardiographic assessment of valvular abnormalities

In the follow-up echocardiography conducted 6 months after the defect closure, the prevalence of valvular abnormalities, including mitral regurgitation (MR), tricuspid regurgitation (TR), and aortic regurgitation (AR) was significantly reduced compared to the baseline echocardiography, and the amount of reduction in the prevalence of atrioventricular valves regurgitation (MR and TR) were more significant than the decline seen in the prevalence of aortic insufficiency (*p*-value < 0.001 for MR and TR vs. *p*-value = 0.033 for AR) (Table 3).

There was no significant difference in the amount of improvement in valvular abnormalities between patients younger and older than 50 (Table 4).

### Complications

Complications of the procedure were more prevalent among individuals aged 50 and older. One patient had moderate pericardial effusion in the anterior part of the right ventricular outflow tract (RVOT), which subsequently resolved through conservative treatment. There was one case of tamponade without cardiac rupture and one case of hematoma accompanied by collapse in the anterior part of the RVOT with a size of 2.8 cm that were both treated successfully by pericardiocentesis. Moreover, a single case of complete atrioventricular (AV) block occurred one month after the procedure and was treated properly by implanting a permanent pacemaker. An isolated case of arteriovenous fistula was also identified at the procedure site, which was closed using a stent graft. In patients younger than 50, there were only two reported cases of mild pericardial effusion that were both resolved by conservative treatment.

There were no reports of residual shunt, arrhythmia, pleural effusion, device embolization, or erosion among the study participants.

## Discussion

According to the existing controversies concerning the benefits of ASD closure in older patients, this study was designed to assess the impact of transcatheter ASD closure on echocardiographic indices and compare the findings between individuals younger and older than 50. This study revealed that ASD closure can lead to a significant decline in the size of four chambers and also SPAP, in addition to a significant improvement in biventricular systolic function, LV diastolic function, and valvular insufficiencies, with the decrement of the RV and bi-atrial sizes, and improvement of the RV systolic and LV diastolic function being significantly more prominent among the patients aged 50 and above.

Our findings are compatible with Salehian et al.’s study on 25 patients who underwent transcatheter ASD device closure and showed a significant improvement in both RV and LV function and a reduction in LA volume [15]. In Fang et al.’s study, the RA size was significantly decreased, and LVEF increased after a 3-month follow-up of 44 patients with ASD device closure [16].

This study demonstrated that the degree of increase in LV size and decrease in LVEF was not significantly different between the two groups. Older patients benefited from transcatheter ASD closure as much as the younger group did regarding LV size and LVEF. However, the improvements in RV size and function, LAVi and RAVi, and LV diastolic function were significantly more prominent in the second group despite these parameters being worse at baseline among these patients. These results align with Khan et al.’s study, which revealed the beneficial effect of ASD closure even in old age (43–92), with a return of RV size and function [11]. In the study by Humenberger et al., RV size was significantly higher in the older group and reduced significantly after ASD closure, and the changes were significant between the age groups (younger than 40 and older than 60) [17]. Stroker and colleagues’ study revealed an increase in LVEF, LVEDD, and E/A ratio and a significant decrease in RV and RA dimensions in patients older than 60 [18]. RVEDD and LVEDD were significantly reduced in all the age groups of patients in the study of Takaya and colleagues, showing that cardiac remodeling improved even in older patients, which is consistent with our findings [12].

This study showed that ASD closure leads to a significant decrement of baseline MR, TR, and AR, with the improvement being more prominent in atrioventricular valves than the aortic valve. There was no significant difference in the level of improvement of valvular abnormalities between subjects younger and older than 50 years old. Our findings regarding TR are compatible with the results of previous studies that demonstrated ASD closure, even in the elderly, can lead to a significant reduction in the severity of TR by decreasing right ventricular preload and improving its geometric abnormality [19, 20, 21, 22]. Yalonetsky et al. compared changes in TR after transcatheter ASD closure in a group of adults (40–59 years old) and elderly (older than 60 years old) and revealed that although at the baseline, TR was more prevalent among the elderly, there were no cases of significant TR in either group one year after the defect closure [23]. Our results concerning MR are consistent with the findings of Yew et al., which reported no cases of new-onset or deterioration of pre-existing MR in a 5-year follow-up of patients with transcatheter ASD closure [24]. In contrast, some previous studies have reported some degrees of MR worsening. Nakagawa et al. reported a slight increase in the degree of MR following transcatheter ASD closure in 37% of the elderly individuals with no impact on their NYHA function class, mainly attributable to the senile degenerative changes in mitral valve leaflets [25]. Wilson et al. reported MR aggravation in 10% of 194 patients [26], and Hiraishi et al. reported it in 37% of patients with transcatheter ASD closure, who were more likely to be older, with larger ASD sizes and smaller horizontal-to-vertical ratio of basal-RV at end-systole [27]. It seems that in older patients with long-term volume and pressure overload due to ASD, the inability of the mitral valve annulus to adapt to the cardiac geometric changes following defect closure may affect the configuration of the mitral annulus and lead to worsening of MR. Other possible explanations for MR aggravation following ASD closure include an increase in the left ventricular preload and elevation of LV filling pressure, atrial malfunction due to the stiffness caused by the occluder device, configurative alterations in the mitral ring, or the impingement of the device on the ring [28]. Our findings about AR are in accordance with the results of Loar et al., that reported percutaneous ASD device closure doesn’t increase the incidence or severity of AR over a mean follow-up of 1.2 years [29] and has a marked difference with Shoen et al. study that reported new-onset or deterioration of AR in 9% of patients who underwent ASD closure, probably as a result of geometric changes in the septum and device-induced traction on the non-coronary aortic valve sinus [30].

In the current study, a significant decline was observed in the median SPAP, and the prevalence of pulmonary hypertension following defect closure, but the extent of changes wasn’t significantly different among the two age groups, and patients aged 50 and older had higher PAPs both at baseline and 6 months after defect closure compared to those younger than 50. Our findings were consistent with the findings of Humenberger and colleagues in 2010 that detected a significant and similar decrease in PAP after ASD closure in all age groups, and older patients with higher PAPs at the baseline ended up with significantly higher PAPs after the defect closure compared to the younger individuals [17]. Yalonetsky et al. also ended up with similar results in their study comparing changes in PAP following transcatheter ASD closure in 2 groups of adults and elderly. He discovered that although both groups experienced a marked reduction in PAP after defect closure and the changes in the elderly group were non-significantly more prominent than the adult group, patients older than 60 had significantly higher PAPs both at baseline and one year after the procedure [23]. Yong et al.’s study also confirms our findings that although patients with more advanced PH experienced a larger extent of reduction in their PAP, they were less likely to reach normal PAP after defect closure [31]. While Gabriels and colleagues found out that as the age at the time of closure advances, especially in those above 55 years old, the risk of persistence of PH or new occurrence of PH despite normal PAP before defect closure increases and the clinical outcome may also be worse compared to those who underwent defect closure in younger ages [32].

Following a 6-month follow-up in this study, none of the patients required surgical repair, and there were no instances of residual shunt during the follow-up period, resulting in a success rate of 100%. ASD closure in patients with LV dysfunction may carry the risk of pulmonary edema due to rapid changes in left-sided pressure [33]. However, in this study, with 63% of participants having systolic dysfunction and 40% with diastolic dysfunction, we did not observe any instances of pulmonary edema in either group. According to Snijder et al.‘s researches, the most common complications of ASD device closure include device embolization, AV block, device thrombosis, and pericardial effusion [34]. In the group aged below 50 years old, only two patients experienced mild pericardial effusion, which was managed through conservative treatment. In the group aged over 50 years, five patients encountered adverse effects, including moderate pericardial effusion, tamponade, complete AV block after 1-month, arteriovenous fistula at the procedure site, and hematoma in the anterior RVOT. These complications were sequentially managed with conservative treatment, pericardiocentesis, permanent pacemaker (PPM) implantation, stent closure, and pericardiocentesis. Adverse effects were more frequent in the individuals aged over 50 years. However, all of these complications were successfully treated and did not result in any mortality.

This study has several limitations that should be acknowledged. First, the study’s retrospective design may introduce inherent biases in data collection and interpretation, limiting the ability to establish causality. Second, the single-center nature of the study and the predominance of female participants may limit the generalizability of our findings. However, since the gender distribution was similar between both age groups, the internal validity of our comparisons remains intact. The COVID-19 pandemic during our study period was another limitation that resulted in a decline in the number of patients undergoing ASD closure at our center due to decreased non-emergency interventions to prioritize patient well-being during the pandemic. Moreover, limited access to patients for extended follow-up posed another limitation attributable to the center’s role as a tertiary facility, attracting patients from various regions of the country. Furthermore, the absence of complete NYHA (New York Heart Association) function class information for all patients presented a constraint, making it difficult to accurately assess their clinical symptoms before and after the intervention. Finally, future multi-center studies with larger sample sizes, more balanced gender distribution, and longer follow-up durations are warranted to validate and extend our findings.

## Conclusion

ASD device closure leads to a significant decline in the size of four chambers and SPAP, in addition to a significant improvement in biventricular function and valvular insufficiencies. Reductions in the bi-atrial and RV dimensions and improvements in the RV systolic and LV diastolic function were significantly more prominent in the individuals aged 50 and older. The findings of this study demonstrate that transcatheter ASD device closure is a beneficial procedure with a high success rate and low complication rate among older individuals, leading to improvements in cardiac form and function.

## Electronic supplementary material

Below is the link to the electronic supplementary material.

Supplementary Material 1

## Data Availability

Data is provided within the manuscript or supplementary information files.

## Abbreviations

AR: Aortic regurgitation
ASD: Atrial septal defect
AV: Atrioventricular
CHD: Congenital heart disease
LAVi: Left atrial volume index
LV: Left ventricle
LVEF: LV ejection fraction
MR: Mitral regurgitation
NYHA: New York Heart Association
PH: Pulmonary hypertension
PPM: Permanent pacemaker
Q_p_/Q_s_: Pulmonary to systemic blood flow ratio
RA: Right atrium
RAVi: Right atrial volume index
RV: Right ventricle
RVOT: Right ventricular outflow tract
SPAP: Systolic pulmonary arterial pressure
TEE: Transesophageal echocardiography
TR: Tricuspid regurgitation
TTE: Transthoracic echocardiography
