# Supplementary material for: Comparing the impact of transcatheter ASD closure on echocardiographic indices in adults below and above 50 years
Source: Echo Res Pract. 2025 May 5;12:10. doi: 10.1186/s44156-025-00074-3 (PMC12051308; doi:10.1186/s44156-025-00074-3)
Supplement: Supplementary file 1 — Supplementary Material 1 [file 44156_2025_74_MOESM1_ESM.docx]

**Supplementary files**

**Comparing the Impact of Trans-catheter ASD Closure on Echocardiographic Indices in Adults and the Elderly**

**Table of contents:**

- **Results:**

Table S1 ………………………………………………………………………………….... Page 2

| **Table S1-** Comparison of baseline and follow-up echocardiographic findings between patients younger and older than 50 years old | | | | | | |
| --- | --- | --- | --- | --- | --- | --- |
|  | **Baseline** | | | **Follow-up** | | |
|  | **Age < 50 years (n=105)** | **Age ≥ 50 years (n=135)** | **P-value** | **Age < 50 years (n=105)** | **Age ≥ 50 years (n=135)** | **P-value** |
| **LV parameters** |  |  |  |  |  |  |
| LV size (mm), median [Q1-Q3] | 45 (40-47) | 45 (40-50) | 0.156 | 40 (37-45) | 40 (37-45) | 0.469 |
| LV size category, n (%) |  |  | 0.832 |  |  | 0.596 |
| Normal | 96 (91%) | 124 (92%) |  | 102 (97%) | 131(97%) |  |
| Mild enlargement | 6 (6%) | 8 (6%) |  | 3 (3%) | 2 (1.5%) |  |
| Moderate enlargement | 3 (3%) | 3 (2%) |  | 0 (0%) | 2 (1.5%) |  |
| LVEF, n (%) |  |  | 0.001 |  |  | 0.014 |
| Normal | 49 (47%) | 37 (27%) |  | 81 (77%) | 84 (62%) |  |
| Mild dysfunction | 53 (50%) | 85 (63%) |  | 24 (23%) | 51 (38%) |  |
| Moderate dysfunction | 3 (3%) | 13 (10%) |  | 0 (0%) | 0 (0%) |  |
| LV diastolic dysfunction, n (%) |  |  | < 0.001 |  |  | 0.123 |
| Normal | 78 (74%) | 61 (45%) |  | 91 (87%) | 108 (80%) |  |
| Mild dysfunction | 23 (22%) | 67 (50%) |  | 14 (13%) | 25 (18.5%) |  |
| Moderate dysfunction | 4 (4%) | 5 (4%) |  | 0 (0%) | 2 (1.5%) |  |
| Severe dysfunction | 0 (0%) | 2 (1%) |  | 0 (0%) | 0 (0%) |  |
| **RV parameters** |  |  |  |  |  |  |
| RV size (mm), median [Q1-Q3] | 33 [30-34] | 35 [30-39] | 0.001 | 30 [29-32] | 30 [27-35] | 0.173 |
| RV size category, n (%) |  |  | < 0.001 |  |  | < 0.001 |
| Normal | 61 (58%) | 51 (38%) |  | 92 (88%) | 86 (63.7%) |  |
| Mild enlargement | 30 (29%) | 34 (25%) |  | 12 (11%) | 40 (29.6%) |  |
| Moderate enlargement | 13 (12%) | 40 (30%) |  | 1 (1%) | 9 (6.6%) |  |
| Severe enlargement | 1 (1%) | 10 (7%) |  | 0 (0%) | 0 (0%) |  |
| RV function, n (%) |  |  | < 0.001 |  |  | 0.799 |
| Normal | 58 (55%) | 33 (24.4%) |  | 81 (77%) | 106 (78.5%) |  |
| Mild dysfunction | 44 (42%) | 87 (64.4%) |  | 24 (23%) | 29 (21.5%) |  |
| Moderate dysfunction | 3 (3%) | 15 (11.1%) |  | 0 (0%) | 0 (0%) |  |
| SPAP (mmHg), median [Q1-Q3] | 32 [30-36] | 35 [31-40] | < 0.001 | 25 [23-30] | 30 [25-35] | < 0.001 |
| SPAP category, n (%) |  |  | 0.001 |  |  | < 0.001 |
| Normal | 69 (66%) | 63 (47%) |  | 97 (92%) | 99 (73%) |  |
| Top normal PH | 22 (21%) | 34 (25%) |  | 5 (5%) | 22 (16%) |  |
| Mild PH | 14 (13%) | 31 (23%) |  | 3 (3%) | 13 (10%) |  |
| Moderate PH | 0 (0%) | 7 (5%) |  | 0 (0%) | 1 (1%) |  |
| **Atrial parameters** |  |  |  |  |  |  |
| LAVi (mL/m2), median [Q1-Q3] | 32 (28-35) | 35 (30-41) | < 0.001 | 30 (25-31) | 30 (27-35) | < 0.001 |
| LA size category, n (%) |  |  | < 0.001 |  |  | < 0.001 |
| Normal | 73 (70%) | 58 (43%) |  | 96 (91%) | 91 (67%) |  |
| Mild enlargement | 31 (29%) | 43 (32%) |  | 9 (9%) | 38 (28%) |  |
| Moderate enlargement | 1 (1%) | 26 (19%) |  | 0 (0%) | 5 (4%) |  |
| Severe enlargement | 0 (0%) | 8 (6%) |  | 0 (0%) | 1 (1%) |  |
| RAVi (mL/m2), median [Q1-Q3] | 27 (25-30) | 33 (29-39) | < 0.001 | 25 (22-26) | 27 (25-30) | < 0.001 |
| RA size category, n (%) |  |  | < 0.001 |  |  | < 0.001 |
| Normal | 72 (68.5%) | 26 (19%) |  | 92 (88%) | 82 (60.7%) |  |
| Mild enlargement | 31 (29.5%) | 57 (43%) |  | 12 (11%) | 40 (29.6%) |  |
| Moderate enlargement | 2 (2%) | 40 (30%) |  | 1 (1%) | 11 (8%) |  |
| Severe enlargement | 0 (0%) | 11 (8%) |  | 0 (0%) | 2 (1.5%) |  |
| **Valvular parameters** |  |  |  |  |  |  |
| MR, n (%) |  |  | 0.982 |  |  | 0.111 |
| No MR | 7 (7%) | 6 (4.4%) |  | 81 (77%) | 115 (85%) |  |
| Mild MR | 88 (84%) | 119 (88.1%) |  | 24 (23%) | 20 (15%) |  |
| Moderate MR | 10 (9%) | 10 (7.4%) |  | 0 (0%) | 0 (0%) |  |
| TR, n (%) |  |  | 0.026 |  |  | 0.055 |
| No TR | 8 (8%) | 7 (5%) |  | 60 (57%) | 61 (45%) |  |
| Mild TR | 83 (79%) | 94 (70%) |  | 45 (43%) | 73 (54%) |  |
| Moderate TR | 14 (13%) | 34 (25%) |  | 0 (0%) | 1 (1%) |  |
| AI, n (%) |  |  | 0.21 |  |  | 0.377 |
| No AI | 95 (90.4%) | 88 (65%) |  | 96 (91%) | 94 (69.6%) |  |
| Mild AI | 9 (8.6%) | 45 (33%) |  | 9 (9%) | 40 (29.6%) |  |
| Moderate and severe AI | 0 (0%) | 2 (2%) |  | 0 (0%) | 1 (0.7%) |  |
| LV: left ventricle, LVEF: left ventricular ejection fraction, RV: right ventricle, SPAP: systolic pulmonary artery presseure, PH: pulmonary hypertension, LAVi: left atrial volume index, LA: left atrium, RAVi: right atrial volume index, RA: right atrium, MR: mitral regurgitation, TR: tricuspid regurgitation, AI: aortic insufficiency | | | | | | |
